# Multimodal Autonomic Biomarkers Predict Phenoconversion in Pure Autonomic Failure

**DOI:** 10.1002/acn3.70140

**Published:** 2025-07-22

**Authors:** S. Koay, E. Vichayanrat, F. Bremner, F. Valerio, R. Mackenzie, G. Chiaro, G. Ingle, P. McNamara, L. Watson, J. N. Panicker, M. P. Lunn, C. Mathias, V. Iodice

**Affiliations:** ^1^ Autonomic Unit National Hospital for Neurology and Neurosurgery London UK; ^2^ Department of Translational Neuroscience and Stroke University College London Queen Square Institute of Neurology London UK; ^3^ Queen Square Centre for Neuromuscular Diseases National Hospital for Neurology and Neurosurgery London UK; ^4^ Neuro‐Ophthalmology Department National Hospital for Neurology and Neurosurgery London UK; ^5^ Department of UroNeurology The National Hospital for Neurology and Neurosurgery London UK

**Keywords:** autonomic failure, autonomic testing, multiple system atrophy, orthostatic hypotension, Parkinson's disease, pure autonomic failure

## Abstract

**Background:**

Pure autonomic failure (PAF) presents with autonomic failure without other neurological features. A third develop central neurological features, fulfilling criteria for multiple system atrophy (MSA) and Lewy body diseases (LBD), including Parkinson's disease and Dementia with Lewy bodies. We hypothesized multimodal autonomic biomarkers would identify differences between PAF, MSA, and LBD, and predict phenoconversion in patients presenting with PAF.

**Methods:**

This observational cohort study included 391 alpha‐synucleinopathy patients evaluated with cardiovascular autonomic testing, plasma noradrenaline, pupillometry, autonomic symptom, and quality‐of‐life questionnaires. PAF patients were monitored for the emergence of central neurological features. Logistic regression modeling was used to identify autonomic biomarkers at initial assessment that predicted future phenoconversion.

**Results:**

Patients with PAF had more severe orthostatic hypotension, lower supine plasma noradrenaline, and frequent sympathetic pupillary deficits at initial assessment than MSA and LBD. 50/194 (26%) with PAF phenoconverted to MSA or LBD after a median of 13 years, with normal pupils, heart rate response to deep breathing ≥ 10 bpm, and supine plasma noradrenaline ≥ 200 pg/mL predicting future phenoconversion to MSA or LBD, with younger age at presentation and higher supine plasma noradrenaline levels associated with conversion to MSA.

**Conclusion:**

In patients presenting with PAF, normal pupillary function and supine plasma noradrenaline levels with intact cardiovagal responses were red flags for future phenoconversion. Younger patients with higher supine plasma noradrenaline levels were more likely to convert to MSA rather than LBD. A non‐invasive multimodal autonomic assessment can help differentiate between alpha‐synucleinopathies and predict phenoconversion from PAF to MSA or LBD.

## 1 Introduction

Alpha‐synucleinopathies are neurodegenerative diseases characterized by the deposition of abnormally phosphorylated α‐synuclein within the central and peripheral nervous system, with varying clinical manifestations including autonomic failure. They include pure autonomic failure (PAF), multiple system atrophy (MSA), Parkinson's disease (PD), and dementia with Lewy bodies (DLB). The 1996 Consensus Criteria define PAF as a sporadic disorder characterized by orthostatic hypotension, typically in the context of more widespread autonomic failure, without other neurological features [1]. Several natural studies have shown that 12%–34% with PAF develop progressive motor and/or cognitive symptoms, fulfilling clinical criteria for MSA, PD, or DLB [2, 3, 4, 5, 6], with a recent study showing abnormal DAT scan in PAF patients up to 7 years before phenoconversion [7], raising debate regarding whether PAF should be considered a prodromal stage of these central nervous system diseases. Identifying early biomarkers to predict which patients with PAF will develop more prominent central nervous system involvement may allow us to intervene with potential disease modifying therapies at a much earlier stage of the disease trajectory.

Neuropathologically, PAF, PD, and DLB are characterized by the accumulation of misfolded α‐synuclein in neuronal cytoplasmic inclusions (Lewy bodies), whereas in MSA, α‐synuclein is primarily deposited in glial cells. Individuals with PAF have predominantly peripheral deposition of α‐synuclein within the sympathetic ganglia and post‐ganglionic neurons and postganglionic autonomic denervation [8]. Supine plasma noradrenaline levels, a reflection of peripheral sympathetic neural activity, are typically low in PAF but normal in MSA [1, 9, 10]. Similarly, cardiac uptake of the adrenergic analogue MIBG (meta‐iodobenzylguanidine) tends to be intact in MSA [11], again suggesting preserved post‐ganglionic sympathetic function, although more recent studies suggest MIBG can be impaired in up to 30% with MSA [12]. Sudomotor studies in individuals with MSA have demonstrated a preganglionic pattern of anhidrosis [13], but more recent, larger studies have also demonstrated post‐ganglionic sudomotor dysfunction and denervation [14, 15, 16], occurring with increasing frequency later in the disease. Overall, these studies suggest the autonomic nervous system can be affected at different levels in α‐synucleinopathies, with predominantly peripheral involvement of the autonomic ganglia and post‐ganglionic neurons in PAF, and predominantly central involvement in MSA, particularly early in the disease.

In the last decade, significant efforts have been directed towards identifying early clinical features and objective biomarkers that are able to predict phenoconversion from PAF to MSA, PD, and DLB. The presence of subtle parkinsonian or cerebellar motor findings on clinical examination is associated with an increased likelihood of conversion to a more widespread α‐synucleinopathy. Higher resting heart rate, greater heart rate rise on tilt, higher Valsalva ratio, higher supine plasma noradrenaline, normal cardiac MIBG, preserved olfaction, early severe bladder symptoms, and catheterisation have been associated with conversion to MSA [3, 4, 5, 17].

Even to experienced clinicians, the overlapping clinical features of the different α‐synucleinopathies at first presentation represent a diagnostic challenge, and the search continues for reliable, non‐invasive biomarkers that can be used to objectively distinguish between these diseases at an early stage. We previously studied and validated a multimodal autonomic testing protocol in a cohort of individuals with autoimmune autonomic ganglionopathy (AAG), demonstrating severe autonomic failure across multiple sympathetic and parasympathetic domains at initial assessment, and objectively quantifying response to immune therapy with longitudinal assessments [18]. We hypothesized deep phenotyping with a multimodal autonomic testing protocol would be able to capture characteristic autonomic phenotypes and identify early biomarkers to differentiate between the α‐synucleinopathies and predict phenoconversion in individuals presenting with PAF to a more widespread α‐synucleinopathy.

We previously defined a composite biomarker, the orthostatic intolerance ratio (OIR), as the change in systolic blood pressure in mmHg divided by the time tolerated on head‐up tilt in minutes, up to a maximum of 10 min [18]. In our previous cohort of patients with AAG, the OIR on head‐up tilt correlated significantly with the severity of orthostatic intolerance and physical limitations reported by patients at baseline, and with improvements reported following immunotherapy, suggesting it was a responsive and relevant quantitative biomarker in this group of patients. In this study, we explored the OIR on passive head‐up tilt (OIR‐tilt) as well as OIR on active stand (OIR‐stand), in patients with α‐synucleinopathies, hypothesizing these would be useful quantitative measures reflecting the severity of orthostatic hypotension in this cohort of patients with neurodegenerative pathology.

## 2 Methods

We retrospectively identified patients seen at a national autonomic referral centre between 1987 and 2021 with a diagnosis of PAF, MSA, and LBD at most recent clinical review, or post‐mortem analysis if available, according to established consensus criteria [1, 2, 19, 20]. All patients underwent a comprehensive initial evaluation from a neurologist with autonomic expertise, cardiovascular autonomic testing, measurement of plasma noradrenaline, pupillometry, and screening for potential secondary causes of autonomic failure, imaging, neurophysiology as required, followed by a multi‐disciplinary discussion regarding likely diagnosis. From 2018, patients were recruited to a prospective natural history study and underwent the assessments above, as well as sudomotor testing, urinary studies, and questionnaires to assess autonomic symptoms and quality of life.

All patients had regular follow‐up assessments to monitor disease progression and screen for the development of additional clinical features, allowing refinement of diagnoses over time. Patients with PAF were only included if they had a follow‐up assessment at least 3 years from first presentation confirming they had not developed any additional neurological features. All prospectively recruited participants provided written informed consent according to the Declaration of Helsinki. The study was approved by the local ethics committee and health research authority and reported in accordance with the STROBE statement for observational studies.

### 2.1 Cardiovascular Autonomic Testing and Plasma Catecholamines

All patients underwent cardiovascular autonomic testing as previously described, with beat‐to‐beat recordings of blood pressure and heart rate in the supine position and with active standing for up to 5 min, head‐up tilt for up to 10 min, isometric exercise, deep breathing, and Valsalva manoeuvre [21]. Some patients developed severe orthostatic hypotension with signs of cerebral hypoperfusion necessitating early termination of stand and/or tilt. OIR‐stand was calculated by dividing the fall in systolic blood pressure (ΔSBP) over the time tolerated on stand in minutes, up to a maximum of 5 min. As before, OIR‐tilt was calculated by dividing the ΔSBP over the time tolerated on head‐up tilt, up to a maximum of 10 min. Valsalva ratio (VR) was calculated by dividing maximum heart rate developing during phase II of the Valsalva manoeuvre over the minimum heart rate occurring within 30 s of the peak heart rate. The blood pressure recovery time (PRT) was defined as the time taken for SBP to recover from phase III of the Valsalva manoeuvre back to the baseline [22]. Blood samples were collected via intravenous forearm catheter at rest and following orthostasis for analysis of plasma noradrenaline using high performance liquid chromatography.

As part of our routine testing protocol, all medications potentially affecting autonomic testing were stopped at least five half‐lives prior to testing, and patients were instructed to consume only water for 4 h prior to testing.

### 2.2 Pupillometry, Sudomotor and Urinary Studies

Infrared pupillometry was used to record baseline pupil diameters and response to light and pharmacological stimulation with topical agents including 0.5% apraclonidine, 1% hydroxyamphetamine, 4% cocaine, and 0.125% pilocarpine. Absent or diminished light reflexes and supersensitivity to dilute pilocarpine were indicative of parasympathetic deficits, and delayed redilation following a light impulse, diminished response to cocaine, or supersensitivity to apraclonidine indicated sympathetic deficits. From May 2019, a prolonged light stimulus was used to assess for pupillary fatigue, a unique phenomenon previously reported only in individuals with ganglionic acetylcholine receptor (gAChR) positive autoimmune autonomic ganglionopathy (AAG) [23].

Patients underwent dynamic sweat testing (DST) at the distal leg bilaterally, an assessment of postganglionic sudomotor function [24]. After iontophoresis with 1% pilocarpine, skin was coated with iodine and the formation of sweat gland imprints on starch covered tape was recorded. Density of activated sweat glands/cm^2^, sweat output/min/cm^2^, and average sweat output/gland were recorded and the average for both sides calculated. Urinary flow when voiding with the sensation of a full bladder was assessed by uroflowmetry (Albany Medical SmartFlow) and post‐void residual (PVR) volume measured using a bladder ultrasound scanner (Bardscan Realtime).

### 2.3 Patient Reported Outcomes

Patient reported outcomes were collected using the abbreviated and refined composite autonomic symptom score (COMPASS‐31), the small fibre neuropathy symptom inventory questionnaire (SFN‐SIQ), and the 36‐item short form health survey (SF‐36). The COMPASS‐31 assesses symptoms in six autonomic domains, with weighted subscores for orthostatic intolerance, vasomotor, secretomotor, gastrointestinal, bladder, and pupillomotor symptoms, giving an overall autonomic symptom score from 0 to 100 [25]. The SFN‐SIQ assesses 12 sensory and autonomic symptoms including sweating, diarrhea, constipation, micturition problems, dry eyes, dry mouth, orthostatic dizziness, palpitations, flushes, skin sensitivity, burning, restless legs, and sheet intolerance of legs, with a score of 0 (never) to 3 (always) given for each section [26]. The SF‐36 assesses eight health concepts: (1) physical limitations, (2) social limitations, (3) role limitations due to physical health, (4) bodily pain, (5) general mental health, (6) role limitations due to emotional problems, (7) vitality, and (8) general health perceptions, with a scoring algorithm generating a range of 0 (worst possible health) to 100 (best possible health) for each domain [27].

### 2.4 Statistical Analysis

Data were captured electronically using a secure Research Electronic Data Capture (REDCap) platform. Statistical analysis was performed using R Studio, Version 1.2.1335. Summary data is displayed as median (interquartile range) for continuous data and numbers (percentages) for categorical data. Distributions of data were assessed for normality by visual inspection and using Shapiro–Wilk tests. Pairwise comparisons were made with unpaired two‐tailed *t*‐tests/Wilcoxon rank‐sum tests, and group comparisons were made with ANOVA/Kruskal–Wallis tests with post hoc comparisons using Tukey/Dunn's tests with Bonferroni corrections as appropriate. Chi‐squared tests were used to compare categorical data. Spearman's rank correlation was used to assess the correlation between linear variables. *p* < 0.05 was considered significant.

### 2.5 Univariate and Multivariate Logistic Regression

Variables were assessed in a univariate then multivariate logistic regression model to determine if they were predictors of a particular final diagnosis. For correlated variables (*p* > 0.40 on Spearman's rank correlation), the variable with the lowest *p*‐value on univariate analysis was used for multivariate analysis.

## 3 Results

We studied a total of 391 patients with α‐synucleinopathies: 146 PAF, 157 MSA (79 predominantly cerebellar features, 65 predominantly Parkinsonian features, 13 mixed features) and 88 LBD (55 PD, 33 DLB). The cohort included 47 patients with MSA confirmed at post‐mortem at the Queen Square Brain Bank [28]. 23 patients (12 MSA, 11 PAF) had phosphorylated‐α‐synuclein deposits visualised on punch skin biopsy as part of a separate research study evaluating the presence of abnormally phosphorylated synuclein (*p*‐syn) on cutaneous somatic and autonomic nerves using indirect immunofluorescence [29]. At first assessment, the MSA group was younger (60 [53–66] years) than the PAF (68 [59–75] years) and LBD groups (71 [65–75] years) (*p* < 0.001). A greater proportion of the LBD group was male (68%) compared to the PAF group (51%, *p* = 0.02, Table 1).

**TABLE 1:** Comparison of cardiovascular autonomic testing, noradrenaline and pupillometry at first assessment in 391 individuals with PAF, MSA and LBD.

	Median, IQR			*p*			
	PAF, *n* = 146	MSA, *n* = 157	LBD, *n* = 88	ANOVA	PAF vs. MSA	PAF vs. LBD	MSA vs. LBD
Age, years	68, 59–75	60, 53–66	71, 65–75	< 0.001	< 0.001	0.01	< 0.001
Male sex, *n*, %	75, 51	93, 58	63, 68	0.04	0.25	0.02	0.19
**Cardiovascular,** ***n***	146	157	88				
Supine							
SBP, mmHg	152, 136–173	141, 123–141	147, 126–168	0.001	0.001	0.18	0.60
HR, bpm	67, 60–73	73, 64–81	65, 59–72	< 0.001	< 0.001	0.73	< 0.001
Head up tilt							
ΔSBP, mmHg	71, 55–90	48, 26–72	49, 31–79	< 0.001	< 0.001	< 0.001	1
ΔHR, bpm	9, 3–17	11, 5–18	11, 7–18	0.23			
OIR‐tilt	8.9, 6.1–17.1	4.8, 2.6–8.2	5.5, 3.1–11	< 0.001	< 0.001	< 0.001	0.24
Iso. exercise							
ΔSBP, mmHg	3, −4 to 9	4, −2‐9	6, 1–13	0.02	1	0.01	0.08
ΔHR, bpm	3, 1–7	4, 2–9	5, 2–9	0.003	0.01	0.02	1
HR_DB_, bpm	3, 0–7	4, 1–7	5, 1–9	0.05			
Valsalva ratio	1.15, 1.07–1.32	1.24, 1.13–1.43	1.23, 1.14–1.37	0.001	0.002	0.04	1
**Noradrenaline,** ***n***	135	118	77				
Supine NA, pg/mL	176, 142–204	263, 222–308	223, 202–254	< 0.001	< 0.001	< 0.001	< 0.001
Δ NA with tilt	10, 2–21	11, 3–45	15, 4–45	0.05			
**Pupillometry,** ***n***	58	25	10				
Normal, n, %	17, 29	17, 68	8, 80	< 0.001	0.002	0.004	0.69
Parasympathetic, n, %	4, 7	1, 4	0, 0	1			
Sympathetic, n, %	37, 64	7, 28	2, 20	0.002	0.01	0.01	1

Abbreviations: HR, heart rate; NA, noradrenaline; SBP, systolic blood pressure.

### 3.1 Cardiovascular Autonomic Testing and Plasma Noradrenaline

All 391 patients underwent cardiovascular autonomic testing. The PAF group had the most severe orthostatic hypotension, with the largest ΔSBP on tilt (71 [55–90] mmHg vs. 48 [26–72] mmHg in MSA and 49 [31–79] mmHg in LBD groups) and OIR‐tilt 8.9 [6.1–17.1] vs. 4.8 [2.6–8.2] in MSA and 5.5 [3.1–11] in LBD groups, *p* < 0.001. They had a lower Valsalva ratio 1.15 [1.07–1.32] vs. 1.24 [1.13–1.43] in MSA and 1.23 [1.14–1.37] in LBD groups, *p* = 0.001, heart rate increment with isometric exercise (3 [1–7] bpm vs. 4 [2–9] bpm in MSA and 5 [2–9] in LBD groups, *p* = 0.003), and heart rate variability with deep breathing (HR_DB_) (3 [0–7] bpm vs. 4 [1–7] bpm in MSA and 5 [1–9] bpm in LBD groups, *p* = 0.05).

330 patients had measurement of plasma noradrenaline in the supine and tilted position. The PAF group had lower supine plasma noradrenaline levels (176 [142–204] pg/mL) compared to MSA (263 [222–308] pg/mL) and LBD groups (223 [202–254] pg/mL) *p* < 0.001. Orthostatic increment of noradrenaline was minimal in all groups, with no significant difference between groups.

### 3.2 Pupillometry

Ninety‐three patients underwent pupillometry. 71% (41/58) with PAF had abnormal pupillary function, compared with only 32% (8/25) MSA and 20% (1/10) with LBD, *p* < 0.001, with sympathetic deficits being the most common abnormality in all groups. None had combined sympathetic and parasympathetic deficits. No patients who underwent testing for pupil fatigue had this finding (11 PAF, 2 MSA).

### 3.3 Prospective Study

From 2018, 52 patients were recruited to our prospective natural history study (28 PAF, 18 MSA, 6 LBD: 5 PD, 1 DLB) and underwent cardiovascular autonomic testing, plasma noradrenaline, and pupillometry as well as sudomotor testing, urinary studies, and autonomic symptom and quality of life questionnaires. At recruitment to the prospective study, the PAF group had a longer disease duration compared to MSA (9 [6–14] years vs. 6 [3–9] years, *p* = 0.04, Table S1).

### 3.4 Cardiovascular Autonomic Testing and Plasma Noradrenaline in Prospective Cohort

Cardiovascular testing (*n* = 52) and plasma noradrenaline (*n* = 49) at recruitment to the prospective study showed similar patterns to the retrospective study. Compared with the MSA group, the PAF group had a greater ΔSBP on standing ([62–95] mmHg vs. 40 [30–77] mmHg) and OIR‐stand (11 [7–27] vs. 6 [4–11], *p* = 0.3), reflecting more severe orthostatic hypotension. Supine plasma noradrenaline levels were lowest in PAF (167 [140–190] pg/mL), highest in MSA (246 [234–303] pg/mL) and intermediate in LBD (216 [158–223] pg/mL, *p* < 0.001).

### 3.5 Sudomotor Testing

Forty‐eight patients had DST performed at the distal leg (26 PAF, 17 MSA, 4 LBD). All groups had abnormal postganglionic sudomotor dysfunction, with reduced sweat output (76 [53–158] nL/cm^2^/min, normal ≥ 417 nL/cm^2^/min), sweat gland density (38 [25–53] sweat glands/cm^2^, normal ≥ 64 glands/cm^2^), and sweat output/gland (2.5 [1.3–3.8] nL/min, normal ≥ 5.6 nL/min), with no differences between groups.

### 3.6 Urinary Studies

Thirty‐five patients had PVR recorded (13 MSA, 22 PAF). Urinary retention with elevated PVR > 100 mL was more common in MSA (11/13, 85%) compared to PAF (7/22, 32%, *p* = 0.02). A third of those with MSA (6/18) used urinary catheters at first assessment, and a further third (6/18) were subsequently referred for clean intermittent self‐catheterisation (CISC) due to persistently elevated PVR (67%, 12/18 in total). Fewer patients with PAF used urinary catheters at first assessment (14%, 4/28), with 21% (6/28) subsequently referred for CISC (35%, 10/28 in total, *p* = 0.04).

Thirty‐two patients had uroflowmetry performed (10 MSA, 22 PAF). Of 10 with MSA who had uroflowmetry, one with overactive bladder symptoms voided a small volume < 100 mL so flow profile could not be analyzed. 7/9 (78%) who voided ≥ 100 mL had an abnormal flow profile. The two patients with normal flow profiles had elevated PVR of 122–156 mL, meaning all MSA patients had abnormal bladder testing. 22 patients with PAF had uroflowmetry. Three patients voided < 100 mL, meaning flow pattern could not be assessed, and of these two were already known to have urinary retention and used CISC. 13/19 (68%) patients voiding ≥ 100 mL had abnormal flow profiles. Among the six with normal flow profiles, only one had elevated ≥ 100 mL (Table S2).

### 3.7 Patient Reported Outcomes

Forty patients completed the COMPASS‐31 autonomic symptom and SFN‐SIQ questionnaires, and 42 patients completed the SF‐36 quality of life questionnaires. The PAF group reported the most severe orthostatic intolerance, with significantly higher orthostatic intolerance subscores than MSA on COMPASS‐31 (32 [28–36] vs. 20 [9–28], *p* = 0.01) and SFN‐SIQ questionnaires (3 [2, 3] vs. 1 [1, 2], *p* = 0.04). On the SF‐36 quality of life questionnaire, most patients reported impaired physical function (25[15–49]), and role limitations due to physical health (0 [0–25]), with no difference between groups (0: severe limitations, 100: no limitations, Table S3). The severity of autonomic symptoms reported on COMPASS‐31 correlated with the severity of orthostatic hypotension on head‐up tilt as measured by OIR‐tilt (*ρ* = 0.32, *p* = 0.04).

### 3.8 Summary of Autonomic Phenotype in α‐Synucleinopathies

Overall, compared to MSA and LBD groups, the PAF group had significantly more severe sympathetic cardiovascular autonomic failure, more frequent pupillary deficits, and lower supine plasma noradrenaline levels at first evaluation, in keeping with postganglionic sympathetic denervation. In contrast, the MSA group was younger at initial assessment, in keeping with a more aggressive neurodegenerative pathology, with higher supine heart rate and supine plasma noradrenaline levels, suggesting relatively preserved postganglionic sympathetic innervation, with a large proportion demonstrating urinary retention with PVR ≥ 100 mL and requiring catheterisation. The LBD group was older, in keeping with a more gradually progressive neurodegenerative disorder, with intermediate supine plasma noradrenaline levels (Table 2).

**TABLE 2:** Summary of significant differences between clinical features, cardiovascular testing, noradrenaline, and pupillometry in PAF, MSA, and LBD groups at first evaluation.

First evaluation	PAF	MSA	LBD
Age < 70 years	+++	+++++	++
Supine heart rate ≥ 70 bpm	++	+++	++
Δ SBP on tilt ≥ 60 mmHg	++++	++	++
OIR‐tilt ≥ 6	++++	++	+++
Valsalva ratio < 1.2	++++	++	+++
Supine noradrenaline ≥ 200 pg/mL	++	+++++	++++
Pupillometry			
Normal	++	++++	++++
Sympathetic deficit	++++	++	+
Parasympathetic deficit	+	+	−

*Note:* +, 1%–20%; ++, 21%–40%; +++, 41%–60%; ++++, 61%–80%; +++++, 81%–100%.

### 3.9 Univariate and Multivariate Logistic Regression Modeling

#### 3.9.1 Variables Distinguishing MSA From PAF

**FIGURE 1:**
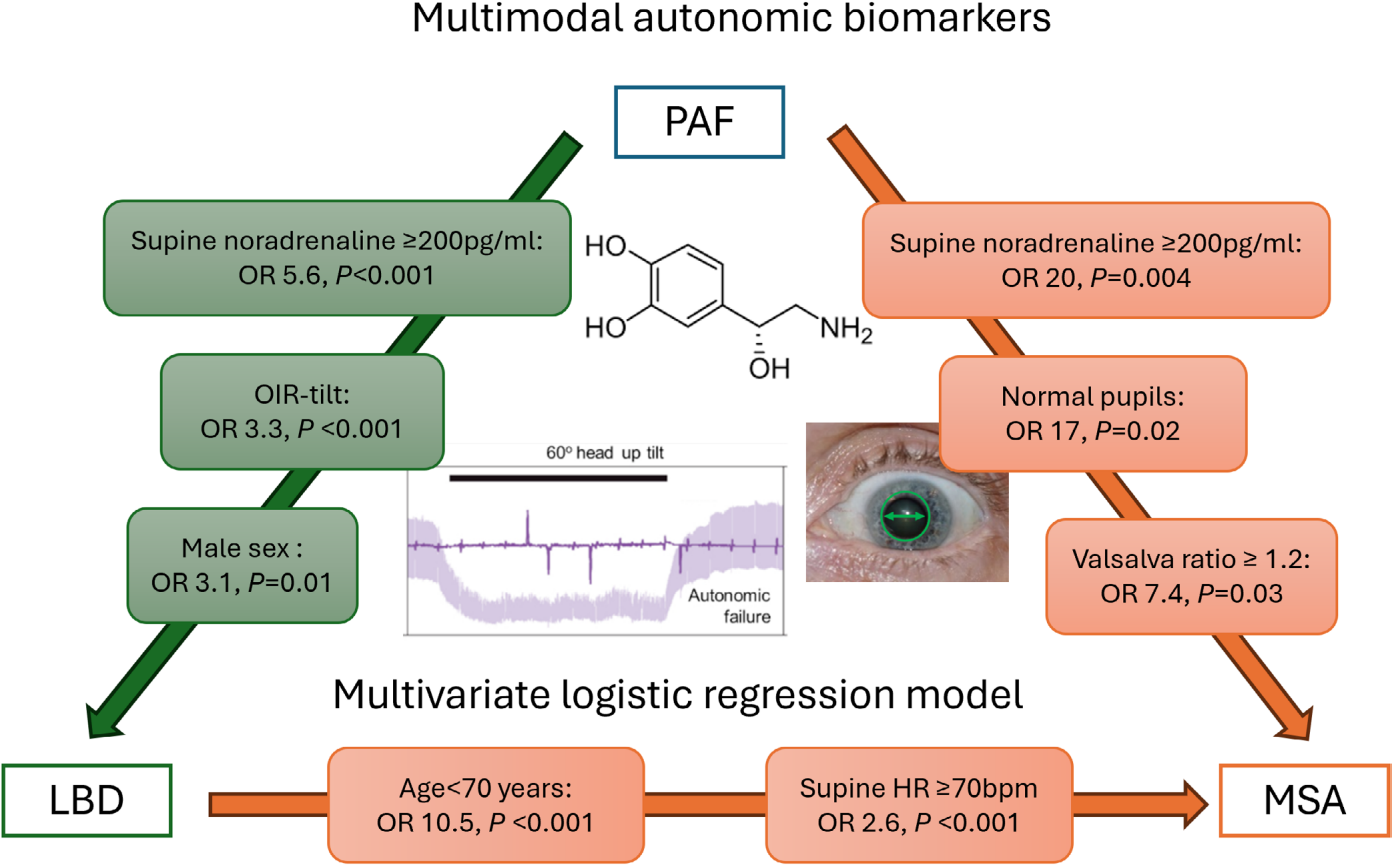
Multimodal autonomic biomarkers independently associated with a final diagnosis of LBD and MSA on multivariate logistic regression modelling.

In a univariate logistic regression model, younger age at first assessment, lower supine SBP, higher supine heart rate, lower ΔSBP on tilt and OIR‐tilt, higher ΔHR on isometric exercise, higher Valsalva ratio, higher supine plasma noradrenaline, and normal pupils were associated with a final diagnosis of MSA rather than PAF. On multivariate analysis, normal supine noradrenaline ≥ 200 pg/mL (OR 20, 95% CI 2–158, *p* = 0.004), normal pupils (OR 17, 95% CI 2–188, *p* = 0.02), Valsalva ratio ≥ 1.2 (OR 7.5, 95% CI 1.3–44.1 *p* = 0.03), and higher supine heart rate (OR 1.4, 95% CI 1.1–1.7, *p* = 0.01) were associated with a final diagnosis of MSA (Table 3, Figure 1).

**TABLE 3:** Variables independently associated with risk on multivariate logistic regression modelling for (A) MSA versus PAF, (B) LBD versus PAF, (C) MSA versus LBD.

Variables on initial assessment	OR, 95% CI	*p*
(A) MSA versus PAF		
Supine noradrenaline ≥ 200 pg/mL	20, 3–158	0.004
Normal pupils	17, 2–188	0.02
Valsalva ratio ≥ 1.2	7.4, 1.3–44.2	0.03
Supine heart rate, bpm	1.3, 1.1–1.7	0.01
(B) LBD versus PAF		
Supine noradrenaline ≥ 200 pg/mL	5.5, 2.9–10.5	< 0.001
OIR‐tilt < 6	3.3, 1.8–6.0	< 0.001
Male sex	3.1, 1.3–7.3	0.01
Age, years	1.07, 1.03–1.12	0.002
(C) MSA versus LBD		
Age < 70 years	10.5, 4.8–22.9	< 0.001
Supine heart rate ≥ 70 bpm	2.6, 1.5–4.4	< 0.001
Supine noradrenaline, pg/mL	1.01, 1.00–1.02	0.001

#### 3.9.2 Variables Distinguishing LBD From PAF

Univariate modeling showed older age, male sex, lower supine SBP, lower ΔSBP and OIR‐tilt, higher ΔHR with isometric exercise, higher Valsalva ratio, higher HR_DB_, higher supine noradrenaline, and normal pupils were associated with a final diagnosis of LBD rather than PAF. Only 10 patients with LBD had pupillometry, so this was omitted from multivariate modeling. Excluding pupillometry, significant predictors in a multivariate model for a final diagnosis of LBD rather than PAF were normal supine noradrenaline levels ≥ 200 pg/mL (OR 5.50, 95% CI 2.89–10.47, *p* < 0.001), OIR‐tilt < 6 (OR 3.26, 95% CI 1.76–6.04, *p* < 0.001), male sex (OR 3.10, 95% CI 1.31–7.30, *p* = 0.01) and older age at first assessment (OR 1.07, 95% CI 1.03–1.12, *p* = 0.002).

#### 3.9.3 Variables Distinguishing MSA From LBD

Univariate and multivariate modeling showed age < 70 years at first assessment (OR 10.5, 4.8–22.9, *p* < 0.001), supine HR ≥ 70 bpm (OR 2.6, 1.5–4.3, *p* < 0.001), and higher supine plasma noradrenaline (OR 1.01, 1.00–1.02, *p* = 0.001) were all significant predictors of a final diagnosis of MSA rather than LBD.

### 3.10 Phenoconversion From PAF to MSA or LBD

**FIGURE 2:**
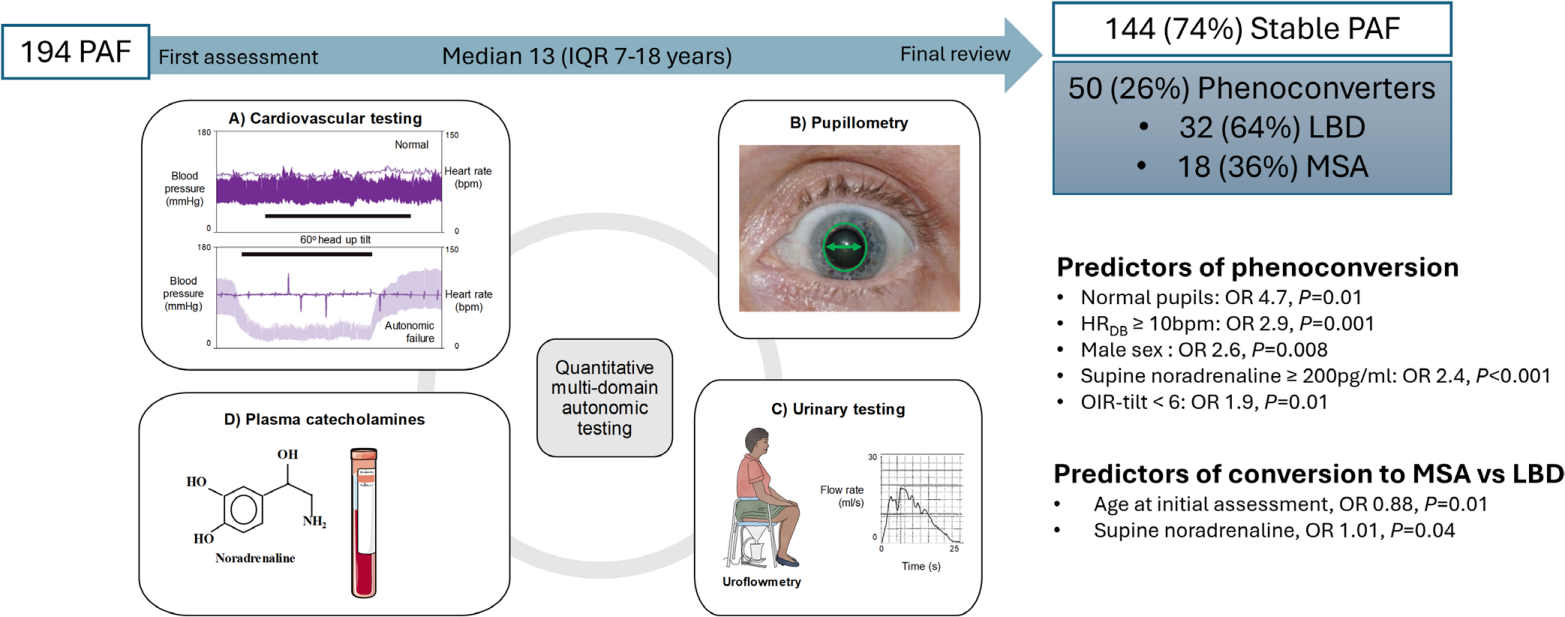
Predictors of conversion from PAF to MSA or LBD on univariate logistic regression modelling.

Having established differences in the autonomic phenotype at initial presentation between PAF, MSA, and LBD, we further investigated whether multimodal autonomic testing could identify objective biomarkers to predict phenoconversion in patients initially presenting with PAF. We examined a subset of 194 patients in our entire cohort with an initial diagnosis of PAF, comparing the autonomic profile at first assessment between patients who retained their PAF at final review and those who subsequently phenoconverted to MSA or LBD, after a median follow‐up of 13 years (IQR 7–18 years). At final review, 144/194 (74%) retained their PAF diagnosis, and 50/194 (26%) had developed signs and symptoms fulfilling the criteria for a more widespread α‐synucleinopathy: 32 LBD, 18 MSA (Figure 2).

#### 3.10.1 Predictors of Phenoconversion

Amongst the subset presenting with PAF, the phenoconverter group had more males (73%) compared to the stable PAF group (51%, *p* = 0.01, Table 4). The phenoconverter group had less severe orthostatic hypotension initial assessment (OIR‐tilt 6.3 [4.5–11.6] vs. 9 [6.2–17.5], *p* = 0.004), more preserved HR_DB_ (4 [1–11] bpm vs. 3 [0–7] bpm, *p* = 0.04), supine noradrenaline levels (210 [161–246] pg/mL vs. 176 [141–204] pg/mL, *p* < 0.001) and pupillary function (67% vs. 30% with normal pupils, *p* = 0.02) (Table 4).

**TABLE 4:** Comparison between individuals with stable PAF and individuals phenoconverting to MSA or LBD.

	Median, IQR		*p*
	Stable PAF, *n* = 144	Phenoconverter, *n* = 50	
Age, years	68, 59–75	67, 60–73	0.59
Male sex, *n*, %	73, 51	37, 73	0.01
**Cardiovascular testing,** ***n***	144	50	
Supine			
SBP, mmHg	152, 136–173	141, 132–162	0.06
HR, bpm	67, 60–74	67, 62–74	0.58
Head up tilt			
ΔSBP, mmHg	71, 56–90	62, 45–86	0.09
ΔHR, bpm	10, 3–18	13, 8–21	0.045
OIR‐tilt	9, 6.2–17.5	6.3, 4.5–11.6	0.003
Isometric exercise			
ΔSBP, mmHg	3, −4 to 9	6, 2–13	0.01
ΔHR, bpm	3, 1–7	4, 2–8	0.22
HR_DB_, bpm	3, 0–6.5	4, 1–11	0.04
Valsalva ratio	1.15, 1.07–1.32	1.21, 1.16–1.35	0.06
**Noradrenaline,** ***n***	134	48	
Supine NA, pg/mL	176, 141–204	210, 161–246	< 0.001
ΔNA on tilt, pg/mL	10, 2–22	9, 2–28	0.97
**Pupillometry,** ***n***	57	15	
Normal, n, %	17, 30	10, 67	0.02
Sympathetic, n, %	36, 63	5, 33	0.046

Abbreviations: HR, heart rate; NA, noradrenaline; SBP, systolic blood pressure.

On univariate logistic regression modeling, normal pupils (OR 4.7, 95% CI 1.2–15.9, *p* = 0.01), HR_DB_ ≥ 10 bpm (OR 2.9, 95% CI 1.5–5.4, *p* = 0.001), male sex (OR 2.6, 95% CI 1.3–5.1, *p* = 0.01), supine noradrenaline ≥ 200 pg/mL (2.4, 95% CI 1.5–3.9, *p* < 0.001), and OIR‐tilt < 6 (OR 1.9, 95% CI 1.17–3.04, *p* = 0.01) predicted future phenoconversion (Table 5). A multivariate analysis was not performed due to the small numbers of converters with all parameters assessed.

**TABLE 5:** Predictors of phenoconversion on univariate logistic regression modeling.

	OR, 95% CI	*p*
Predictors of phenoconversion in individuals with PAF		
Normal pupils	4.7, 1.4–15.9	0.01
HR_DB_ ≥ 10 bpm	2.9, 1.5–5.4	0.001
Male sex	2.6, 1.3–5.2	0.008
Supine noradrenaline ≥ 200 pg/mL	2.4, 1.5–3.9	< 0.001
OIR‐tilt < 6	1.9, 1.8–3.0	0.01
Predictors of conversion to MSA rather than LBD		
Age at initial assessment, years	0.88, 0.81–0.97	0.01
Supine noradrenaline, pg/mL	1.01, 1.00–1.03	0.04

#### 3.10.2 Predictors of Conversion to MSA Versus LBD

The MSA converter group was younger at first assessment than the LBD converter group (59 [50–67] years vs. 71 [64–74] years, *p* = 0.001), with higher supine plasma noradrenaline (242 [201–316] pg/mL vs. 204 [159–228] pg/mL, *p* = 0.04). On univariate and multivariate analysis, younger age at first assessment (OR 0.88, 95% CI 0.81–0.97, *p* = 0.01) and higher supine plasma noradrenaline (OR 1.01, 95% CI 1.00–1.03, *p* = 0.04) predicted phenoconversion to MSA rather than LBD (Table 5).

## 4 Discussion

We present the largest study evaluating multimodal autonomic biomarkers in α‐synucleinopathies at a national autonomic unit over 30 years. Previous functional and pathological studies in α‐synucleinopathies have demonstrated the multifocal nature of synuclein deposition and dysfunction within the central and peripheral autonomic nervous system [8, 30, 31, 32, 33]. Multimodal objective biomarkers to assess the central and peripheral autonomic pathways and characterize the extent and severity of autonomic dysfunction in this cohort may improve diagnostic accuracy at an earlier stage of presentation. Compared to MSA, the PAF group had more severe orthostatic hypotension, more frequent sympathetic pupillary deficits, and lower supine noradrenaline levels, in keeping with more severe post‐ganglionic sympathetic dysfunction. In individuals presenting with PAF, less severe orthostatic hypotension, normal pupillary function, and preserved supine plasma noradrenaline levels at initial assessment predicted subsequent phenoconversion to a more widespread α‐synucleinopathy, with younger age and higher supine plasma noradrenaline levels predicting conversion to MSA rather than LBD. Previous studies have identified promising novel biomarkers for differentiating α‐synucleinopathies and predicting phenoconversion in PAF, including MIBG, DATscan (dopamine transporter scan), CSF α‐synuclein levels using real‐time quaking‐induced conversion (RT‐QuIC) and cutaneous phosphorylated α‐synuclein deposits on skin biopsy [12, 29, 30, 32, 34, 35, 36, 37, 38, 39, 40]. The CSF and cutaneous synuclein studies are of particular interest, allowing confirmation of the underlying α‐synuclein pathology in vivo, but are currently limited to specialist centres within a research setting. Incorporating non‐invasive autonomic assessments with a combination of cardiovascular, pupillary, and blood‐based biomarkers into routine evaluations could enhance early diagnostic accuracy in α‐synucleinopathies.

26% of our cohort initially diagnosed with PAF later phenoconverted to either MSA or LBD by the end of the study period, consistent with previous reports [3, 4, 5, 17]. Normal pupils, HR_DB_ > 10 bpm, supine plasma noradrenaline > 200 pg/mL, and OIR‐tilt < 6 on initial assessment predicted future phenoconversion, with younger age and higher supine plasma noradrenaline predicting conversion to MSA rather than LBD. These findings are in agreement with previous natural history studies that found patients converting to MSA/LBD had less severe ΔSBP on tilt on initial assessment [4], and patients converting to MSA rather than LBD were younger at first presentation [17], with higher supine noradrenaline [3, 5, 17]. Importantly, our study is the first to systematically evaluate pupillary function in a large cohort with autonomic failure with longitudinal follow‐up. We found that normal pupils are relatively uncommon in individuals with PAF (29%) compared to MSA and LBD (68%–80%), and normal pupils were associated with 4.7 × increased odds of phenoconversion from PAF to a more widespread α‐synucleinopathy (*p* = 0.01). We would advocate incorporating pupillometry as a non‐invasive and well‐tolerated test in the standard assessment of all patients presenting with autonomic failure. Validating our current pupillometry protocol with handheld pupillometers would enable bedside assessments to be performed as part of the standard work‐up in more disabled patients with severe motor symptoms or severe orthostatic intolerance that may find it challenging to undergo an additional visit to the neuro‐ophthalmology clinic.

In our study, PAF patients had the largest ΔSBP on head‐up tilt compared to MSA and LBD, consistent with Mabuchi et al.'s previous study [9]. In keeping with their objective testing, PAF patients reported more severe orthostatic intolerance, but similarly severe impairment in daily activities and role limitations due to physical health compared to MSA. Even in the absence of motor symptoms, severe autonomic failure is associated with significant physical disability, and warrants prompt recognition, assessment, and treatment. When assessing orthostatic intolerance, we know some individuals with severe orthostatic hypotension cannot tolerate a full 10‐min head‐up tilt challenge, as they have a precipitous fall in blood pressure with symptoms of cerebral hypoperfusion and need to be returned supine within a few minutes. In these individuals, the change in systolic blood pressure alone underrepresents the severity of orthostatic intolerance. We previously explored the OIR‐tilt in patients with AAG, a composite measure combining both ΔSBP and time tolerated on tilt, and found it was a sensitive biomarker that correlated with severity of patient‐reported orthostatic intolerance [18]. In this much larger cohort with α‐synucleinopathies, we found that patients with an initial diagnosis of PAF, while ΔSBP on head‐up tilt was not significantly different between the phenoconverter and stable PAF subgroups (*p* = 0.09), OIR‐tilt was significantly lower in the phenoconverter group (*p* = 0.004) and OIR‐tilt < 6 was a risk factor for future phenoconversion on univariate logistic regression (OR 1.9, *p* = 0.01). Patients in our prospective cohort also had OIR‐stand calculated with a 5‐min active standing challenge as well as head‐up tilt, with significant differences in OIR‐stand between the PAF and MSA groups. This is of particular interest as a 5‐min active standing challenge can be easily performed in a non‐specialist outpatient setting.

Previous pathological studies have shown predominantly peripheral ganglionic and post‐ganglionic α‐synuclein deposition in PAF in the form of Lewy Bodies, whereas in MSA there is more abundant central α‐synuclein deposition in the form of glial inclusions in the brain and spinal cord [41]. Mabuchi et al.'s previous study found individuals with PAF had lower supine plasma noradrenaline, suggesting greater postganglionic sympathetic denervation compared to individuals with MSA [9]. Lipp et al.'s previous study did not find any significant differences in supine plasma noradrenaline levels or orthostatic increment between MSA and PD [13]. To the best of our knowledge, our study has the largest cohort with α‐synucleinopathies with plasma noradrenaline levels reported to date. We found supine noradrenaline levels were significantly different between all three groups of α‐synucleinopathies, with the lowest levels in PAF, highest levels in group, and intermediate levels in LBD. Normal supine plasma noradrenaline ≥ 200 pg/mL in patients presenting with PAF was associated with a 2.4× increased odds of phenoconverting to MSA or LBD, with higher levels associated with phenoconversion to MSA rather than LBD (*p* < 0.001) [3, 5, 17].

We found higher supine heart rate in MSA compared to PAF and LBD, in keeping with previous cardiac scintigraphy studies that have shown patients with MSA have a relatively intact cardiac sympathetic innervation compared to PAF and LBD [9, 42, 43]. We also found patients with PAF had the lowest Valsalva ratio and heart rate increment with isometric exercise, and lower HR_DB_ compared to MSA and LBD, suggesting more abnormal sympathetic as well as parasympathetic cardiovagal innervation. Both HR_DB_ and ΔHR on head‐up‐tilt ≥ 10 bpm predicted phenoconversion in PAF, suggesting that early cardiac autonomic denervation is a key feature of PAF, and relatively intact heart rate responses should be a red flag for future conversion.

A significantly higher proportion of individuals with MSA had elevated PVR > 100 mL and required catheterisation compared to individuals with PAF, consistent with previous studies describing severe, early urinary dysfunction in MSA and occurring as a late feature in PAF [9]. Nevertheless, it is worth noting that almost a third of individuals with PAF had urinary retention with PVR > 100 mL at first assessment, and of those with PVR < 100 mL, 9/14 (64%) had an abnormal flow pattern, of whom two went on to develop persistently elevated PVR requiring catheterisation. Our data suggest that urinary dysfunction is not an uncommon feature in PAF, who should all have a basic bladder assessment, ideally with uroflowmetry and PVR. Individuals with persistently elevated PVR ≥ 100 mL should be referred for CISC training, and individuals with abnormal uroflowmetry without urinary retention at initial assessment should have interval studies to monitor for development of urinary retention.

### 4.1 Limitations

Our cohort is derived from referrals to a national autonomic unit, who are more likely to have severe autonomic symptoms due to referral bias. In particular, the patients referred with PD tend to be those with prominent autonomic features or other atypical features, meaning our findings may not be representative of the more typical patient with idiopathic Parkinson's disease.

## 5 Conclusions

Patients with α‐synucleinopathies present with a spectrum of clinical features indicating involvement of different structures within the central and peripheral autonomic nervous system over the course of the disease. We advocate the use of non‐invasive multimodal objective assessments to quantify the severity of autonomic failure across multiple autonomic domains, localize the predominant pattern of autonomic dysfunction, and gain early insights into the underlying pathology. Our study suggests in PAF, the characteristic combination of normal pupillary function, normal supine plasma noradrenaline levels, with relatively less severe orthostatic hypotension and preserved heart rate responses is concerning for future phenoconversion, with younger age at presentation and higher supine plasma noradrenaline levels associated with conversion to MSA rather than LBD. With promising advancements in potential disease‐modifying treatment in these neurodegenerative diseases, early diagnosis and recruitment of patients in the prodromal or premotor phase is paramount. The addition of multimodal non‐invasive quantitative assessments within a neuroscience centre can help to facilitate an early diagnosis, and future studies that validate methods of performing these assessments with hand‐held or bedside equipment would further improve the accessibility and convenience for patients with neurodegenerative diseases.

## Author Contributions

S.K., E.V., F.B., and V.I. contributed to the conception and design of the study. S.K., E.V., F.B., F.V., R.M., G.C., and L.W. contributed to the acquisition and analysis of data. S.K., E.V., F.B., G.I., P.M., J.N.P., M.P.L., C.M., and V.I. contributed to drafting a significant portion of the manuscript or figures.

## Conflicts of Interest

V.I. has received honoraria from Theravance Biopharma not related to this work. The other authors have no potential conflicts of interest to declare relevant to the research in the submitted manuscript.

## Supporting information


**Table S1:** Cardiovascular autonomic testing and plasma catecholamines in prospective cohort.
**Table S2:** Bladder studies in prospective cohort.
**Table S3:** Autonomic symptoms and quality of life questionnaires in prospective cohort.

## Data Availability

The data that support the findings of this study are available from the corresponding author upon reasonable request.
