# Supplementary material for: Multimodal Autonomic Biomarkers Predict Phenoconversion in Pure Autonomic Failure
Source: Ann Clin Transl Neurol. 2025 Jul 22;12(11):2170–80. doi: 10.1002/acn3.70140 (PMC12623832; doi:10.1002/acn3.70140)
Supplement: Supplementary file 1 — Table S1: Cardiovascular autonomic testing and plasma catecholamines in prospective cohort. Table S2: Bladder studies in prospective cohort. Table S3: Autonomic symptoms and quality of life questionnaires in prospective cohort. [file ACN3-12-2170-s001.docx]

**Supplementary Table 1.** Cardiovascular autonomic testing and plasma catecholamines in prospective cohort

|  | Median, IQR | | | *P*-value | | | |
| --- | --- | --- | --- | --- | --- | --- | --- |
|  | PAF, n=28 | MSA, n=18 | LBD, n=6 | ANOVA | PAF vs MSA | PAF vs LBD | MSA vs LBD |
| Demographics |  |  |  |  |  |  |  |
| Male, n, % | 17, 68 | 12, 66 | 5, 83 | .83 |  |  |  |
| Age, y | 61, 57-69 | 63, 54-67 | 71, 66-73 | .24 |  |  |  |
| Disease dur, y | 9, 6-14 | 6, 3-9 | 6, 5-10 | .047 | .04 | 1 | 1 |
|  |  |  |  |  |  |  |  |
| Cardiovascular autonomic testing | | | | | | | |
| Supine |  |  |  |  |  |  |  |
| SBP, mmHg | 151, 142-170 | 133, 118-152 | 161, 131-188 | .03 | .03 | 1 | .42 |
| HR, bpm | 66, 58-73 | 70, 61-76 | 67, 65-67 | .66 |  |  |  |
| Stand |  |  |  |  |  |  |  |
| Δ SBP, mmHg | 81, 62-95 | 40, 30-77 | 80, 49-102 | .03 | .03 | .88 | .40 |
| Δ HR, bpm | 7, 3-16 | 11, 4-17 | 17, 13-20 | .44 |  |  |  |
| OIR-stand | 18, 13-25 | 8, 6-17 | 16, 10-21 | .03 | .03 | .65 | 1 |
| Tilt |  |  |  |  |  |  |  |
| Δ SBP, mmHg | 76, 57-97 | 62, 40-78 | 74, 41-105 | .32 |  |  |  |
| Δ HR, bpm | 1, -7 to 18 | 7, 0-14 | 13, -3 to 23 | .64 |  |  |  |
| OIR-tilt | 11, 7-27 | 6, 4-11 | 8, 4-17 | .06 |  |  |  |
| Isometric exercise |  |  |  |  |  |  |  |
| Δ SBP, mmHg | 0.5, -5 to 0 | 4, -2 to 9 | 5, -2 to 17 | .40 |  |  |  |
| Δ HR, bpm | 3, 1-6 | 5, 3-7 | 10, 6-14 | .07 |  |  |  |
| PRT, s | 23, 17-29 | 17, 6-26 | 13, 10-16 | .07 |  |  |  |
| Valsalva ratio | 1.22, 1.11-1.37 | 1.20, 1.13-1.34 | 1.14, 1.10-1.22 | .77 |  |  |  |
| HR_DB_ | 4.5, 0-8 | 4, 0.5-7 | 4, 4-6 | .98 |  |  |  |
|  |  |  |  |  |  |  |  |
| Noradrenaline, n | 28 | 16 | 5 |  |  |  |  |
| Supine, pg/ml | 167, 140-190 | 246, 234-303 | 216, 158-223 | 2 e-8 | <.001 | .19 | .02 |
| Δ NA-tilt, pg/ml | 4, 1-13 | 11, 5-13 | 38, 28-67 | .02 | .53 | .02 | .32 |
|  |  |  |  |  |  |  |  |
| Dynamic sweat testing, distal leg, n | 26 | 17 | 5 |  |  |  |  |
| Sweat output, nL/cm^2^/min | 75, 45-137 | 72, 55-155 | 107, 96-166 | .47 |  |  |  |
| Sweat glands/cm^2^ | 38, 27-53 | 37, 20-51 | 47, 34-53 | .62 |  |  |  |
| Sweat output/gland, nL/cm^2^/min | 2.1, 1.2-3.3 | 2.5, 1.3-4.3 | 2.9, 2.9-3.8 | .61 |  |  |  |

*SBP, systolic blood pressure; HR, heart rate; NA, noradrenaline.*

**Supplementary Table 2.** Bladder studies in prospective cohort

|  | PAF, n=28 | MSA, n=18 | *P*-value |
| --- | --- | --- | --- |
|  |  |  |  |
| Post-void residual volume measured, n | 22 | 13 |  |
| Post-void residual volume, ml | 52, 3-131 | 173, 142-250 | .01 |
| Post-void residual volume >100ml, n, % | 7, 32 | 10, 83 | .02 |
|  |  |  |  |
| Uroflow performed, n | 22 | 10 |  |
| Volume voided >100ml, n | 19 | 9 |  |
| Uroflow abnormal, n, % | 13, 68 | 7, 78 | .68 |
|  |  |  |  |
| Urinary catheterisation documented, n | 28 | 18 |  |
| At first assessment, n, % | 4, 14 | 6, 33 | .16 |
| During study, n, % | 10, 35 | 12, 67 | .04 |

**Supplementary Table 3.** Autonomic symptoms and quality of life questionnaires in prospective cohort

|  | Median, IQR | | | *P*-value | | | |
| --- | --- | --- | --- | --- | --- | --- | --- |
|  | PAF  n=28 | MSA  n=18 | LBD  n=6 | ANOVA | PAF vs MSA | PAF vs LBD | MSA vs LBD |
|  |  |  |  |  |  |  |  |
| COMPASS-31, n | 24 | 14 | 2 |  |  |  |  |
| Total | 48, 42-60 | 37, 29-47 | 20, 19-21 | .01 | .10 | .06 | .57 |
| Orthostatic intol. | 32, 28-36 | 20, 9-28 | 10, 5-15 | .002 | .01 | .06 | 1 |
| Vasomotor | 0, 0-0 | 0, 0-0 | 0, 0-0 | .77 |  |  |  |
| Secretomotor | 4.3, 0-8.6 | 4.3, 0-6.4 | 2.1, 1.1-3.2 | .76 |  |  |  |
| Gastrointestinal | 7.6, 5.1-12.5 | 8.0, 5.8-10.5 | 5.8, 3.8-7.8 | .77 |  |  |  |
| Bladder | 3.3, 0.8-5.6 | 3.9, 2.2-6.4 | 0.6, 0.3-0.8 | .10 |  |  |  |
| Pupillomotor | 2, 1.0-2.4 | 2.3, 0.2-3.0 | 1.2, 0.6-1.8 | .75 |  |  |  |
|  |  |  |  |  |  |  |  |
| SFN-SIQ, n | 25 | 12 | 3 |  |  |  |  |
| Total | 16, 9-18 | 10, 8-10 | 12, 8-16 | .04 | .03 | .71 | .75 |
| Sweating abnormalities | 3, 1-3 | 0, 0-2 | 3, 1.5-3 | .05 | .04 | 1 | .61 |
| Diarrhoea | 1, 0-1 | 0.5, 0-1 | 0, 0-0.5 | .33 |  |  |  |
| Constipation | 1, 1-2 | 1, 1-2.3 | 1, 0.5-2 | .70 |  |  |  |
| Micturition problems | 2, 1-3 | 2, 1.8-3 | 1, 0.5-2 | .57 |  |  |  |
| Dry eyes | 0, 0-1 | 0, 0-0 | 0, 0-1.5 | .15 |  |  |  |
| Dry mouth | 1, 0-3 | 0, 0-1.3 | 2, 1-2.5 | .16 |  |  |  |
| Orthostatic dizziness | 3, 2-3 | 1, 1-2.3 | 3, 1.5-3 | .04 | .04 | 1 | .88 |
| Palpitations | 1, 0-2 | 0, 0-0.3 | 0, 0-0 | .05 |  |  |  |
| Hot flashes | 0, 0-1 | 0, 0-0 | 0, 0-1 | .41 |  |  |  |
| Sensitive skin | 0, 0-1 | 0, 0-0 | 0, 0-0 | .05 |  |  |  |
| Burning feet | 0, 0-0 | 0, 0-0 | 0, 0-0.5 | .90 |  |  |  |
| Bedsheet intolerance legs | 0, 0-.8 | 0, 0-0 | 0, 0-0 | .40 |  |  |  |
| Restless legs | 0, 0-2 | 0, 0-1 | 1, 0.5-1.5 | .80 |  |  |  |
|  |  |  |  |  |  |  |  |
| SF-36, n | 25 | 15 | 2 |  |  |  |  |
| Physical function | 25, 20-50 | 15, 5-30 | 45, 25-65 | .21 |  |  |  |
| Role limitations, physical | 0, 0-25 | 0, 0-25 | 50, 25-75 | .71 |  |  |  |
| Role limitations, emotional | 67, 33-100 | 67, 33-100 | 50, 25-75 | .83 |  |  |  |
| Energy/fatigue | 35, 25-45 | 40, 30-50 | 33, 29-36 | .55 |  |  |  |
| Emotional well-being | 48, 40-76 | 36, 32-60 | 76, 70-82 | .18 |  |  |  |
| Social functioning | 50, 38-63 | 38, 13-63 | 75, 69-81 | .08 |  |  |  |
| Pain | 58, 33-90 | 70, 41-90 | 61, 47-76 | .93 |  |  |  |
| General health | 50, 30-65 | 50, 23-58 | 28, 26-29 | .36 |  |  |  |
